# ﻿Novel *Helicoma* and *Neohelicosporium* (Tubeufiaceae, Tubeufiales) species and two new host records of *Helicoma* on tropical palms (Arecaceae) from China

**DOI:** 10.3897/mycokeys.108.128889

**Published:** 2024-09-13

**Authors:** Yinru Xiong, Kevin D. Hyde, Li Lu, Dulanjalee L. Harishchandra, Ausana Mapook, Biao Xu, Fatimah Alotibi, Ishara S. Manawasinghe

**Affiliations:** 1 Innovative Institute for Plant Health, Zhongkai University of Agriculture and Engineering, Guangzhou 510225, Guangdong, China; 2 School of Science, Mae Fah Luang University, Chiang Rai 57100, Thailand; 3 Center of Excellence in Fungal Research, Mae Fah Luang University, Chiang Rai 57100, Thailand; 4 CAS Key Laboratory for Plant Diversity and Biogeography of East Asia, Kunming Institute of Botany, Chinese Academy of Science, Kunming, China; 5 Center for Yunnan Plateau Biological Resources Protection and Utilization, College of Biological Resource and Food Engineering, Qujing Normal University, Qujing, Yunnan 655011, China; 6 Office of Research Administration, Chiang Mai University, Chiang Mai 50200, Thailand; 7 Department of Entomology and Plant Pathology, Faculty of Agriculture, Chiang Mai University, Chiang Mai 50200, Thailand; 8 Department of Botany and Microbiology, College of Science, King Saud University, P.O. Box 22452, 11495 Riyadh, Saudi Arabia

**Keywords:** *Caryotamitis*, *Elaeisguineensis*, *Elaeisoleifera*, helicosporous fungi, phylogeny, saprobic fungi, taxonomy, three new species

## Abstract

Asexual species of Tubeufiaceae are characterised as helicosporous hyphomycetes and are abundantly discovered in tropical and subtropical regions. The present study collected helicosporous fungal samples from rotting tissues of *Caryotamitis*, *Elaeisguineensis* and *E.oleifera* in Xishuangbanna, Yunan Province, China. Fungal isolates were identified, based on the morphological characteristics and multi-gene phylogeny with DNA sequence data of the internal transcribed spacer (ITS), part of the large subunit nuclear rRNA gene (LSU), translation elongation factor 1-alpha gene (*tef* 1-*α*) and RNA polymerase II second largest subunit gene (*rpb*2). Herein, we introduce three new species viz. *Helicomaoleifera*, *Neohelicosporiumguineensis* and *N.xishuangbannaensis*. In addition, we introduce two new host records of *Helicomaguttulatum* and *H.rufum* on *Caryotamitis*. The illustrations of all identified species, detailed descriptions and in-depth phylogenetic analyses are provided. Our results add new knowledge of fungal species associated with palm hosts in southern China. Moreover, our data will contribute to the biodiversity of fungi in tropical China.

## ﻿Introduction

Regions in southern China exhibit characteristics of a monsoon climate and its weather patterns are additionally influenced by the geographical distribution and differentiation of land and sea (Wang et al. 1999; Peng et al. 2021). Therefore, the boundaries of China’s tropics are long and incoherent (Gongfu et al. 1990). In this fragmented tropical region spanning from south-eastern to south-western China, the flora shows certain differences depending on the geographical composition in different regions (Zhu 2017). There are 23 large plant families containing 100–200 species of tropical flora in different regions, amongst which 101 species and 18 genera are from Arecaceae (Zhu 2016, 2017).

Arecaceae species are commonly known as palms and they are common in tropical evergreen forests. These species are available in every ecological habitat in the Tropics and Sub-tropics and regulate the composition and climate in those ecosystems (Reichgelt et al. 2018; Fehr et al. 2020). They are rich in fungal diversity covering most major groups of fungi and have been widely reported and studied (Fröhlich and Hyde 1999, 2000; Taylor et al. 1999; Konta et al. 2023; Pereira and Phillips 2023). Amongst these, many Tubeufiaceae species are recorded in the history of studies of palm fungi (Pereira and Phillips 2023). A few examples are: Pirozynski (1972) reporting *Helicomaambiens* on oil palm from Tanzania, *Aquaphilaramdayalea* reported by Bhat (2008) on *Caryotaurens* from India and *Berkleasmiumcorticola* reported by Capdeet and Romero (2010) on *Butiayatay* and *Syagrusromanzoffiana* from Argentina.

Tubeufiaceae was introduced by Barr (1979), based on the type genus *Tubeufia* to accommodate bitunicate ascomycetes occurring as saprobes on decaying wood. Tubeufiaceae has fascinating and peculiar morphs of both sexual and asexual morphs (Zhao et al. 2007; Li et al. 2022). These species are prevalently distributed in temperate and tropical regions (Rossman 1987; Kirk et al. 2001; Lumbsch and Huhndorf 2010; Boonmee et al. 2014; Luo et al. 2017; Lu et al. 2018a, b). Although members of this family can be found in both terrestrial woody substrates and in aquatic habitats, an interesting phenomenon is that most asexual morphs of Tubeufiaceae are collected from freshwater habitats (Hyde et al. 2016, 2017; Brahamanage et al. 2017; Chaiwan et al. 2017; Lu et al. 2017a, b, c, 2018a, b; Liu et al. 2018; Hongsanan et al. 2020). The distinguishing characteristic of Tubeufiaceae is the asexual morph mostly found as helicosporous hyphomycetes, while some contain phragmosporous and chlamydosporous conidia (Lu et al. 2018b; Dong et al. 2020). Their sexual morphs are characterised by superficial ascomata, bitunicate asci and ascospores which are hyaline to pale brown, elongate, obovoid or oblong and septate (Barr 1980; Kodsueb et al. 2006; Boonmee et al. 2011, 2014; Brahamanage et al. 2017; Lu et al. 2018b). Following Wijayawardene et al. (2022) and Ma et al. (2023), 47 genera have been accepted in Tubeufiaceae including *Helicoma* and *Neohelicosporium*.

*Helicoma* was proposed by Corda (1837), with *H.muelleri* as the type species. It is one of the earliest genera of helicosporous hyphomycetes (Linder 1929; Moore 1955; Goos 1986; Boonmee et al. 2014; Lu et al. 2018b). *Helicoma* species are frequently reported as saprobes inhabiting terrestrial and aquatic environments (Zhao et al. 2007; Boonmee et al. 2011, 2014; Hyde et al. 2016; Lu et al. 2018b, 2023; Liu et al. 2019; Tian et al. 2022). Based on phylogenetic analysis of combined ITS, LSU, *tef* 1-*α* and *rpb*2, Lu et al. (2018b) accepted species that are different from typical-Helicoma morphs described by Goos (1986) as *Helicoma*. These species are characterised by conidiogenous cells that are intercalary, cylindrical, with denticles, arising laterally from the lower portion of conidiophores as tooth-like protrusions. Conidia are pleurogenous, helicoid, hygroscopic, tapering towards the apex and rounded at the tip, coiled 1½–5 times, becoming loosely coiled in water. Subsequently, *H.hydei* (Liu et al. 2019), *H.wuzhishanense* (Lu et al. 2022), *H.acropleurogenum* and *H.liyui* (Lu et al. 2023) were reported in this genus.

*Neohelicosporium* was introduced by Lu et al. (2018a) to accommodate taxa with special helicosporous spores based on morphology and phylogenetic analysis of combined ITS, LSU, *tef* 1-*α*, and *rpb*2. Their unique characteristics include branched or unbranched conidiophores arising from creeping hyphae, mono- to polyblastic, integrated, sympodial conidiogenous cells with denticles and acrogenous and/or acropleurogenous conidia, which are used to distinct *Neohelicosporium* from *Helicosporium* (Lu et al. 2018b). After synonymising several species from *Helicoma*, *Helicomyces*, *Helicosporium*, and *Tubeufia*, 25 species are accepted in *Neohelicosporium* (Lu et al. 2018b; Index Fungorum 2024).

To explore the relationship between Tubeufiaceae associated with various palms, the present study collected terrestrial decaying samples of *Caryotamitis*, *Elaeisguineensis* and *E.oleifera*. A total of 12 isolates were obtained, from which we introduce three new species: *Helicomaoleifera*, *Neohelicosporiumguineensis* and *N.xishuangbannaensis* and two new host records: *Helicomaguttulatum* and *H.rufum*. Species descriptions, illustrations of macroscopic and microscopic morphology and phylogenetic analyses are provided to delineate new and known species.

## ﻿Materials and methods

### ﻿Samples collection and isolation

Samples were collected in 2023 from an unidentified forest area beside National Highway 219 in Xishuangbanna in Yunnan Province, China (21°93'20"N, 101°24'57"E, 549.6 m elev.). These samples were rotting materials from different palm species namely, *Caryotamitis*, *Elaeisoleifera* and *E.guineensis*. Samples were brought into the laboratory using plastic ziplock bags and relevant macro and micro-characteristics were photographed by a ZEISS SteREO Discovery V20 stereomicroscopy (Germany) and Nikon Eclipse 80i and the industrial DigitaL Sight DS-Fi1 (Panasonic, Japan) microscope. Following the methods of Senanayake et al. (2018, 2020), single-spore isolation was performed. Germinated spores were aseptically transferred into fresh potato dextrose agar (PDA) plates and incubated at 25 °C to obtain pure cultures (Senanayake et al. 2020). The cultures obtained during the study were deposited in the culture collection of Zhongkai University of Agriculture and Engineering (ZHKUCC). Herbarium materials were deposited at the Mycological Herbarium of Zhongkai University of Agriculture and Engineering (MHZU). Facesoffungi (FoF) numbers and Index Fungorum (IF) numbers were obtained as explained in Jayasiri et al. (2015) and Index Fungorum (2024).

### ﻿Morphological characterisation

After the fungal samples were brought into the laboratory, the Cnoptec SZ650 series (Cnoptec, China) stereomicroscope was used to observe the macromorphological characteristics and photographs were taken using SteReo Discovery V20. Nikon Eclipse 80i and the industrial DigitaL Sight DS-Fi1 (Panasonic, Japan) microscope and imaging system were used to take pictures of micromorphological characters. Digital images of micromorphological structures, including shape, size and colour were recorded. The measurement of structures, including spore dimensions for each species was conducted using NIS-Elements BR 5.30.03. Adobe Photoshop CC 2019 and Adobe Illustrator CC 2019 software (Adobe Systems Inc., San Jose, America) were used to develop images and make photo plates. All pure cultures obtained in this study were grown on potato dextrose agar (PDA) at 25 °C in 12 hours of daylight for a week and the diameter of the culture was measured after six weeks. AxioVersion Rel. 4.8 was used to take photos of the cultures.

### ﻿DNA extraction, PCR amplification and sequencing

The pure cultures were cultured on PDA plates for 1–2 weeks and about 500 mg of fresh fungal mycelia were scraped. Total genomic DNA was extracted from the mycelia using MagPure Plant AS Kit (Magen Biotech, China) following the manufacturer’s instructions. Four nuclear gene regions: internal transcribed spacer (ITS), large subunit nuclear rRNA gene (LSU), translation elongation factor 1-*α* (*tef* 1-*α*) and RNA polymerase II second largest subunit gene (*rpb*2) were amplified using the primers shown in Table 1. The PCR reaction mixture contained 25 μl of total volume, which consisted of 12.5 μl 2 × FastTaq Premix (mixture of FastTaq TM DNA Polymerase, buffer, dNTP Mixture and stabiliser) (Beijing Qingke Biological Technology Co., Ltd., Beijing, PR China), 1 μl of forward and reverse primers each, 9.5 μl ddH_2_O and 1 μl DNA. The polymerase chain reaction (PCR) was performed in a C1000 TouchTM thermal cycler. The PCR procedure is as follows: for ITS/LSU, the initial denaturation step is performed at 95 °C for 2 minutes, then 35 amplification cycles at 95 °C for 1 minute, 50 °C for 1 minute and 72 °C for 1 minute. Finally, extension for 10 minutes at 72 °C. For *tef* 1-*α*, an initial step of 2 minutes at 95 °C followed by 35 cycles of 1 minute at 95 °C, 1 minute at 52 °C, 1 minute at 72 °C and 7 minutes at 72 °C. For *rpb*2 PCR conditions, an initial denaturation step was performed at 95 °C for 5 minutes and then 30 cycles at 94 °C for 1 minute, 53 °C for 30 seconds, 72 °C for 90 seconds and finally 72 °C for 10 minutes. After PCR amplification, the product was observed on a 1% agarose gel under ultraviolet light. DNA sequencing was completed in Tianyi (Guangzhou, China) Co., Ltd. New sequences are deposited in GenBank. The sequences used for analyses with accession numbers are given in Table 2.

**Table 1.** Genes and corresponding primers used in this study.

Gene	Primer	Sequence (5’-3’)	Reference
ITS	ITS5	GGAAGTAAAAGTCGTAACAAGG	White et al. (1990)
	ITS4	TCCTCCGCTTATTGATATGC	
LSU	LR0R	ACCCGCTGAACTTAAGC	Vilgalys and Hester (1990)
	LR5	TCCTGAGGGAAACTTCG	
*tef* 1-*α*	EF1-983F	GCYCCYGGHCAYCGTGAYTTYAT	Carbone and Kohn (1999)
	EF1-2218R	TACTTGAAGGAACCCTTACC	
*rpb*2	fRPB2-5F	GAYGAYMGWGATCAYTTYGG	O’Donnell et al. (2007)
	RPB2-7cr	CCCATRGCTTGYTTRCCCAT	

**Table 2.** Taxon names, strain numbers and corresponding GenBank accession numbers of the taxa used in the Tubeufiaceae phylogenetic analyses.

Species	Strain numbers	ITS	LSU	*tef* 1-α	*rpb*2
*Acanthohelicosporaaurea*	GZCC 16-0060	KY321323	KY321326	KY792600	MF589911
*Acanthohelicosporapinicola*	MFLUCC 10-0116	KF301526	KF301534	KF301555	NA
*Aquaphilaalbicans*	BCC 3543	DQ341096	DQ341101	NA	NA
*Aquaphilaalbicans*	MFLUCC 16-0010	KX454165	KX454166	KY117034	MF535255
*Berkleasmiumfusiforme*	MFLUCC 17-1979	MH558694	MH558821	MH550885	MH551008
*Berkleasmiumlongisporum*	MFLUCC 17-1990	MH558697	MH558824	MH550888	MH551011
*Botryosphaeriaagaves* ^T^	MFLUCC 10-0051	JX646790	JX646807	JX646855	NA
*Botryosphaeriadothidea*	CBS 115476	NA	NG_027577	NA	NA
*Chlamydotubeufiacylindrica* ^T^	MFLUCC 16-1130	MH558702	MH558830	MH550893	MH551018
*Chlamydotubeufiahuaikangplaensis* ^T^	MFLUCC 10-0926	JN865210	JN865198	NA	NA
*Chlamydotubeufiakrabiensis* ^T^	MFLUCC 16-1134	KY678767	KY678759	KY792598	MF535261
*Dematiohelicomyceshelicosporus* ^T^	MFLUCC 16-0213	KX454169	KX454170	KY117035	MF535258
*Dematiohelicomyceshelicosporus*	MFLUCC 16-0003	MH558703	MH558831	MH550894	MH551019
*Helicoarctatusaquaticus* ^T^	MFLUCC 17-1996	MH558707	MH558835	MH550898	MH551024
*Helicodochiumaquaticum*	MFLUCC 16-0008	MH558708	MH558836	MH550899	MH551025
*Helicodochiumaquaticum* ^T^	MFLUCC 17-2016	MH558709	MH558837	MH550900	MH551026
*Helicohyalinumaquaticum* ^T^	MFLUCC 16-1131	KY873625	KY873620	KY873284	MF535257
*Helicohyalinuminfundibulum* ^T^	MFLUCC 16-1133	MH558712	MH558840	MH550903	MH551029
*Helicomaacropleurogenum* ^T^	GZCC 22-2035	OP806857	OP806854	OP821894	OP821897
*Helicomaambiens*	UAMH 10533	AY916451	AY856916	NA	NA
*Helicomaambiens*	UAMH 10534	AY916450	AY856869	NA	NA
*Helicomaaquaticum* ^T^	MFLUCC 17-2025	MH558713	MH558841	MH550904	MH551030
*Helicomabrunneisporum* ^T^	MFLUCC 17-1983	MH558714	MH558842	MH550905	MH551031
*Helicomadennisii*	NBRC 30667	AY916455	AY856897	NA	NA
*Helicomafreycinetiae* ^T^	MFLUCC 16-0363	MH275062	MH260295	MH412770	NA
*Helicomafusiforme* ^T^	MFLUCC 17-1981	MH558715	NA	MH550906	NA
*Helicomaguttulatum* ^T^	MFLUCC 16-0022	KX454171	KX454172	MF535254	MH551032
*Helicomaguttulatum*	GZCC 22-2004	OP508739	OP508779	OP698090	OP698079
*Helicomaguttulatum*	GZCC 22-2024	OP508733	OP508773	OP698084	OP698073
*Helicomaguttulatum*	GZCC 22-2025	OP508737	OP508777	OP698088	OP698077
*Helicomaguttulatum*	MFLUCC 21-0152	OL545456	OL606150	OL964521	OL964527
***Helicomaguttulatum***	**ZHKUCC 24-0139**	**PP860094**	**PP860106**	**PP858054**	**PP858066**
***Helicomaguttulatum***	**ZHKUCC 24-0140**	**PP860095**	**PP860107**	**PP858055**	**PP858067**
*Helicomahongkongense*	MFLUCC 17-2005	MH558716	MH558843	MH550907	MH551033
*Helicomahydei*	MFLUCC 18-1270	MH747101	MH747116	MH747100	NA
*Helicomainthanonense* ^T^	MFLUCC 11-0003	JN865211	JN865199	NA	NA
*Helicomakhunkornensis* ^T^	MFLUCC 10-0119	JN865203	JN865191	KF301559	NA
*Helicomalinderi*	NBRC 9207	AY916454	AY856895	NA	NA
*Helicomaliyui*	GZCC 22-2033	OP806858	OP806855	OP821895	NA
*Helicomalongisporum*	GZCC 22-2005	OP508740	OP508780	OP698091	OP698080
*Helicomalongisporum*	MFLUCC 16-0211	MH558719	MH558845	MH550910	MH551036
*Helicomalongisporum* ^T^	MFLUCC 17-1997	MH558720	MH558846	MH550911	MH551037
*Helicomamiscanthi* ^T^	MFLUCC 11-0375	KF301525	KF301533	KF301554	NA
*Helicomamuelleri*	CBS 964.69	AY916453	AY856877	NA	NA
*Helicomamuelleri*	UBC F13877	AY916452	AY856917	NA	NA
*Helicomamultiseptatum* ^T^	GZCC 16-0080	MH558721	MH558847	MH550912	MH551038
*Helicomanematosporum* ^T^	MFLUCC 16-0011	MH558722	MH558848	MH550913	MH551039
***Helicomaoleifera* ^T^**	**ZHKUCC 24-0121**	**PP860086**	**PP860098**	**PP858056**	**PP858068**
***Helicomaoleifera***	**ZHKUCC 24-0122**	**PP860087**	**PP860099**	**PP858057**	**PP858069**
***Helicomaoleifera***	**ZHKUCC 24-0766**	**PP860088**	**PP860100**	**PP858058**	**PP858070**
***Helicomaoleifera***	**ZHKUCC 24-0767**	**PP860089**	**PP860101**	**PP858059**	**PP858071**
*Helicomarubriappendiculatum* ^T^	MFLUCC 18-0491	MH558723	MH558849	MH550914	MH551040
*Helicomarufum* ^T^	MFLUCC 17-1806	MH558724	MH558850	MH550915	NA
***Helicomarufum***	**ZHKUCC 24-0143**	**PP860096**	**PP860108**	**PP858060**	**PP858072**
***Helicomarufum***	**ZHKUCC 24-0144**	**PP860097**	**PP860109**	**PP858061**	**PP858073**
*Helicomarugosum*	GZCC 22-2034	OP806859	OP806856	OP821896	NA
*Helicomarugosum*	ANM 196	GQ856138	GQ850482	NA	NA
*Helicomarugosum*	ANM 953	GQ856139	GQ850483	NA	NA
*Helicomarugosum*	ANM 1169	NA	GQ850484	NA	NA
*Helicomarugosum*	JCM 2739	NA	AY856888	NA	NA
*Helicomaseptoconstrictum*	MFLUCC 17-1991	MH558725	MH558851	MH550916	MH551041
*Helicomaseptoconstrictum* ^T^	MFLUCC 17-2001	MH558726	MH558852	MH550917	MH551042
*Helicomasiamense* ^T^	MFLUCC 10-0120	JN865204	JN865192	KF301558	NA
*Helicoma* sp.	HKUCC 9118	NA	AY849966	NA	NA
*Helicomatectonae* ^T^	MFLUCC 12-0563	KU144928	KU764713	KU872751	NA
*Helicomavaccinii*	CBS 216.90	AY916486	AY856879	NA	NA
*Helicomawuzhishanense*	GZCC 22-2003	OP508732	OP508772	OP698083	OP698072
*Helicomyceschiayiensis* ^T^	BCRC FU30842	LC316604	NA	NA	NA
*Helicomyceshyalosporus*	MFLUCC 17-0051	MH558731	MH558857	MH550922	MH551047
*Helicomycestorquatus*	MFLUCC 16-0217	MH558732	MH558858	MH550923	MH551048
*Helicosporiumflavum* ^T^	MFLUCC 16-1230	KY873626	KY873621	KY873285	NA
*Helicosporiumluteosporum* ^T^	MFLUCC 16-0226	KY321324	KY321327	KY792601	MH551056
*Helicosporiumvesicarium* ^T^	MFLUCC 17-1795	MH558739	MH558864	MH550930	MH551055
*Helicotruncatumpalmigenum*	NBRC 32663	AY916480	AY856898	NA	NA
*Helicotruncatumpalmigenum*	KUMCC 21-0474	OM102542	OL985959	OM355488	OM355492
*Helicotubeufiaguangxiensis* ^T^	MFLUCC 17-0040	MH290018	MH290023	MH290028	MH290033
*Helicotubeufiahydei* ^T^	MFLUCC 17-1980	MH290021	MH290026	MH290031	MH290036
*Helicotubeufiajonesii* ^T^	MFLUCC 17-0043	MH290020	MH290025	MH290030	MH290035
*Kamalomycesmangrovei*	MFLUCC 17-0407	MH878781	MH878779	MH886508	NA
*Kamalomycesthailandicus*	MFLUCC 13-0233	MF506884	MF506882	MF506886	NA
*Muripulchraaquatica*	KUMCC 15-0245	KY320533	KY320550	KY320563	MH551057
*Muripulchraaquatica*	KUMCC 15-0276	KY320534	KY320551	KY320564	MH551058
*Neoacanthostigmafusiforme* ^T^	MFLUCC 11-0510	KF301529	KF301537	NA	NA
*Neochlamydotubeufiafusiformis* ^T^	MFLUCC 16-0016	MH558740	MH558865	MH550931	MH551059
*Neochlamydotubeufiakhunkornensis*	MFLUCC 16-0025	MH558742	MH558867	MH550933	MH551061
*Neohelicomycesaquaticus*	KUMCC 15-0463	KY320529	KY320546	KY320562	MH551065
*Neohelicomycesgrandisporus* ^T^	KUMCC 15-0470	KX454173	KX454174	NA	MH551067
*Neohelicomycessubmersus* ^T^	MFLUCC 16-1106	KY320530	KY320547	NA	MH551068
*Neohelicosporiumabuense*	CBS 101688	AY916470	NA	NA	NA
*Neohelicosporiumacrogenisporum* ^T^	MFLUCC 17-2019	MH558746	MH558871	MH550937	MH551069
*Neohelicosporiumaquaticum* ^T^	MFLUCC 17-1519	MF467916	MF467929	MF535242	MF535272
*Neohelicosporiumastrictum* ^T^	MFLUCC 17-2004	MH558747	MH558872	MH550938	MH551070
*Neohelicosporiumaurantiellum*	ANM 718	GQ856140	GQ850485	NA	NA
*Neohelicosporiumbambusicola* ^T^	MFLUCC 21-0156	OL606157	OL606146	OL964517	OL964523
*Neohelicosporiumellipsoideum* ^T^	MFLUCC 16-0229	MH558748	MH558873	MH550939	MH551071
*Neohelicosporiumfluviatile*	MFLUCC 15-0606	NA	OP377957	OP473050	OP473111
*Neohelicosporiumfusisporum* ^T^	MFUCC 16-0642	MG017612	MG017613	MG017614	NA
*Neohelicosporiumgriseum*	CBS 961.69	AY916474	AY856884	NA	NA
*Neohelicosporiumgriseum*	CBS 113542	AY916475	AY916088	NA	NA
*Neohelicosporiumguangxiense*	GZCC 16-0042	MF467920	MF467933	MF535246	MF535276
*Neohelicosporiumguangxiense*	MFLUCC 17-0054	MH558750	MH558875	MH550941	MH551073
***Neohelicosporiumguineensis* ^T^**	**ZHKUCC 24-0113**	**PP860090**	**PP860102**	**PP858062**	**PP858074**
***Neohelicosporiumguineensis***	**ZHKUCC 24-0114**	**PP860091**	**PP860103**	**PP858063**	**PP858075**
*Neohelicosporiumhyalosporum* ^T^	GZCC 16-0076	MF467923	MF467936	MF535249	MF535279
*Neohelicosporiumhyalosporum*	GZCC 16-0063	MH558751	MH558876	MH550942	MH551074
*Neohelicosporiumirregulare* ^T^	MFLUCC 17-1796	MH558752	MH558877	MH550943	MH551075
*Neohelicosporiumirregulare*	MFLUCC 17-1808	MH558753	MH558878	MH550944	MH551076
*Neohelicosporiumkrabiense* ^T^	MFLUCC 16-0224	MH558754	MH558879	MH550945	MH551077
*Neohelicosporiumlaxisporum* ^T^	MFLUCC 17-2027	MH558755	MH558880	MH550946	MH551078
*Neohelicosporiummorganii*	CBS 281.54	AY916468	AY856876	NA	NA
*Neohelicosporiummorganii*	CBS 222.58	AY916469	AY856880	NA	NA
*Neohelicosporiumovoideum* ^T^	GZCC 16-0064	MH558756	MH558881	MH550947	MH551079
*Neohelicosporiumovoideum*	GZCC 16-0066	MH558757	MH558882	MH550948	MH551080
*Neohelicosporiumpanacheum*	CBS 257.59	AY916471	AY916087	NA	NA
*Neohelicosporiumparvisporum*	GZCC 16-0078	MF467924	MF467937	MF535250	MF535280
*Neohelicosporiumparvisporum*	MFLUCC 17-2010	MH558763	MH558888	MH550954	MH551086
*Neohelicosporium* sp.	CBS 189.95	AY916472	AY856882	NA	NA
*Neohelicosporium* sp.	HKUCC 10235	NA	AY849942	NA	NA
*Neohelicosporiumsuae*	CGMCC 3.23541	OP184079	OP184068	OP186052	OP265702
*Neohelicosporiumsubmersum*	MFLUCC 17-2376	MT627738	MN913738	NA	NA
*Neohelicosporiumtaiwanense* ^T^	BCRC FU30841	LC316603	NA	NA	NA
*Neohelicosporiumthailandicum* ^T^	MFLUCC 16-0221	MF467928	MF467941	MF535253	MF535283
***Neohelicosporiumxishuangbannaensis* ^T^**	**ZHKUCC 24-0119**	**PP860092**	**PP860104**	**PP858064**	**PP858076**
***Neohelicosporiumxishuangbannaensis***	**ZHKUCC 24-0120**	**PP860093**	**PP860105**	**PP858065**	**PP858077**
*Neotubeufiakrabiensis* ^T^	MFLUCC 16-1125	MG012031	MG012024	MG012010	MG012017
*Parahelicomycesaquaticus* ^T^	MFLUCC 16-0234	MH558766	MH558891	MH550958	MH551092
*Parahelicomyceschiangmaiensis* ^T^	MFLUCC 21-0159	OL697884	OL606145	OL964516	OL964522
*Parahelicomycestalbotii*	MFLUCC 17-2021	MH558765	MH558890	MH550957	MH551091
*Pleurohelicosporiumhyalinum* ^T^	GZCC 20-0489	OP377816	OP377915	OP472996	OP473089
*Pleurohelicosporiumparvisporum* ^T^	MFLUCC 17-1982	MH558764	MH558889	MH550956	MH551088
*Pseudohelicoongigantisporum*	BCC 3550	AY916467	AY856904	NA	NA
*Pseudohelicoonsubglobosum* ^T^	BCRC FU30843	LC316607	LC316610	NA	NA
*Thaxteriellopsislignicola*	MFLUCC 10-0123	JN865207	JN865195	KF301562	NA
*Thaxteriellopsislignicola*	MFLUCC 10-0124	JN865208	JN865196	KF301561	NA
*Tubeufiaabundata* ^T^	MFLUCC 17-2024	MH558769	MH558894	MH550961	MH551095
*Tubeufiaaquatica* ^T^	MFLUCC 16-1249	KY320522	KY320539	KY320556	MH551142
*Tubeufiabambusicola* ^T^	MFLUCC 17-1803	MH558771	MH558896	MH550963	MH551097
*Tubeufiachlamydospora* ^T^	MFLUCC 16-0223	MH558775	MH558900	MH550967	MH551101
*Tubeufiacocois* ^T^	MFLUCC 22-0001	OM102541	OL985957	OM355486	OM355491
*Tubeufiasympodilaxispora* ^T^	MFLUCC 17-0048	MH558808	MH558932	MH551001	MH551135

Ex-type strains are indicated by T after the species name. Newly-generated sequences are indicated in bold. The “NA” indicates information unavailable.

### ﻿Phylogenetic analyses

The quality of the DNA sequences was checked from their chromatograms and the sequences generated by forward and reverse primers were combined using Geneious Prime v. 2021.0.3 (Biomatters Ltd., San Diego, CA, USA). The BLASTn tool (Basic Local Alignment Search Tool) in the search engine of the National Center for Biotechnology Information (NCBI) to analyse the sequences is used in this study (https://blast.ncbi.nlm.nih.gov/Blast.cgi). Based on the BLASTn results, we identified that our isolates belong to *Helicoma* and *Neohelicosporium*. Phylogenetic analyses for Tubeufiaceae were performed following Yang et al. (2023). The sequences for the phylogenetic analysis were downloaded from GenBank and listed in Table 2. MAFFT v. 7 (https://mafft.cbrc.jp/alignment/server/) was used to align and adjust the sequence datasets of the four gene regions. BioEdit 7.0.9.0 was used to improve the alignment manually when necessary. Using Alignment Transformation Environment online (https://sing.ei.uvigo.es/ALTER/), files were converted to run phylogenetic trees. Phylogenetic analysis was conducted using Maximum Likelihood (ML) inferred in RAxML v. 8.2.12 (Stamatakis 2014), Maximum Parsimony (MP) implied on PAUP v. 4.0b10 (Swofford 2003) and Bayesian Inference (BI) on MrBayes v. 3.1.2 (Huelsenbeck and Ronquist 2001).

Maximum parsimony analysis was performed in PAUP (phylogenetic analysis using parsimony) v.4.0b10 (Swofford 2003) using the heuristic search option with tree bisection-reconnection (TBR) branch swapping and 1,000 random sequence additions. Ambiguous regions in the alignment were excluded and gaps were treated as missing data. The stability of the trees was evaluated by 1,000 bootstrap replications. Branches of zero length were collapsed and all multiple parsimonious trees were saved. Descriptive statistics, including tree length (TL), consistency index (CI), retention index (RI), relative consistency index (RC) and homoplasy index (HI) were calculated.

Maximum Likelihood analyses were accomplished using RAxML-HPC2 on XSEDE v. 8.2.8 (Stamatakis et al. 2008; Stamatakis 2014) in the CIPRES Science Gateway platform (Miller et al. 2010) using the GTR+I+G model of evolution with 1,000 non-parametric bootstrapping iterations. MrBayes v.3.0b4 (Huelsenbeck and Ronquist 2001) used for the Bayesian analyses, implemented in MrMTgui (Nuin 2007), was used to determine the best-fit evolution model for Bayesian Inference analyses using the Akaike Information Criterion (AIC). The Markov Chain Monte Carlo sampling (BMCMC) analysis was conducted with four simultaneous Markov chains. The best model of evolution determined for LSU, ITS, *rpb*2 and *tef* 1-*α* by MrModelTest v. 2.2 was GTR+I+G. They were run for 1,000,000 generations, sampling the trees at every 100^th^ generation. From the 10,000 trees obtained, the first 2,000 representing the burn-in phase were discarded. The remaining 8,000 trees were used to calculate posterior probabilities in the majority rule consensus tree. The phylogenetic tree was visualised in FigTree v. 1.4.2. Taxonomic novelties were submitted to the Facesoffungi database (Jayasiri et al. 2015), Index Fungorum (http://www.indexfungorum.org) and Palm Fungi (Xiong et al. 2024) databases. Species delineation was based on criteria set by Chethana et al. (2021) and Pem et al. (2021).

## ﻿Results

### ﻿Phylogenetic analyses

**Figure 1.**
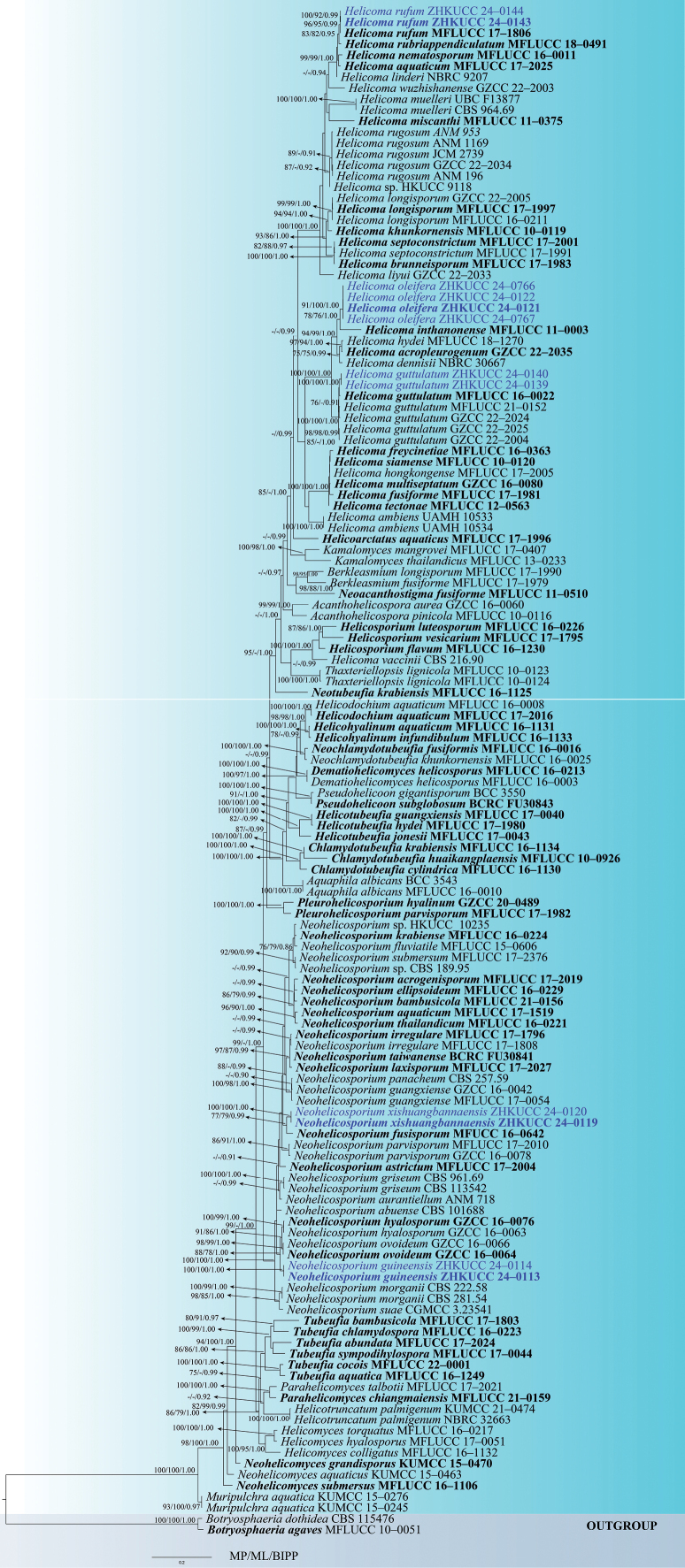
Maximum Likelihood majority rule consensus tree for Tubeufiaceae using ITS, LSU, *rpb*2 and *tef* 1-*α* sequence dataset with *Botryosphaeriaagaves* (MFLUCC 10-0051) and *B.dothidea* (CBS 115476) as the outgroup taxa. Bootstrap support for Maximum Likelihood (ML) and Maximum Parsimony (MP) equal to or greater than 75% and Bayesian Inference posterior probability (BIPP) equal to or greater than 0.90 are indicated above branches as MP/ML/BIPP. The scale bar indicates 0.2 nucleotide changes per site. Isolates from this study are marked in blue and ex-type strains are marked in bold.

Phylogenetic trees were generated by ML, MP and BI of combined ITS (971 bp), LSU (1,172 bp), *rpb*2 (1,045 bp) and *tef* 1-*α* (912 bp) sequence data. The tree topologies generated by these three methods were similar and close to the topology of Yang et al. (2023); the best-scoring ML tree is shown in Fig. 1. The sequence alignment comprised 139 taxa of representative strains of Tubeufiaceae, including 12 isolates obtained in this study. *Botryosphaeriaagaves* (MFLUCC 10-0051) and *B.dothidea* (CBS 115476) were used as the outgroup taxa. Maximum parsimony analysis consisted of 2,223 constant characters and 1,628 informative characters resulting in 368 equally parsimonious trees (Fig. 1) (CI = 0.293, RI = 0.752, RC = 0.220, HI = 0.707). The best-scoring ML tree had an optimisation likelihood value of -51369.027254. The matrix had 2,120 distinct alignment patterns with a 32.53% proportion of gaps and completely undetermined characters. Estimated base frequencies were as follows: A = 0.245833, C = 0.250339, G = 0.261605, T = 0.242223; substitution rates: AC = 1.153811, AG = 5.616698, AT = 2.094588, CG = 0.769981, CT = 8.981963, GT = 1.0; gamma distribution shape parameter α = 0.237020. Incomplete portions at the ends of the sequences were excluded from the analysis. Our 12 new isolates are distributed in five clades, of which eight were distributed in *Helicoma* and four were distributed in *Neohelicosporium*. Based on the phylogenetic evidence and morphology, here we introduce three novel species and two new host records.

### ﻿Taxonomy

#### ﻿ Helicoma oleifera

Taxon classificationFungiTubeufialesTubeufiaceae

Y.R. Xiong, Manawas. & K.D. Hyde sp. nov.

0364608D-CFE2-5BF0-979D-99E43E0C5F94

Index Fungorum: IF902153

Facesoffungi Number: FoF15911

Fig. 2

##### Etymology.

Species epithet refers to the host species name “*oleifera*” from which the fungus was isolated.

##### Holotype.

MHZU 23-0157.

##### Description.

***Saprobic*** on the rotting petiole of *Elaeisoleifera*. ***Sexual morph***: Not observed. ***Asexual morph***: Hyphomycetous, helicosporous. *Colonies* on the substratum superficial, effuse, gregarious, brown. ***Mycelium*** composed of partly immersed, partly superficial, hyaline, septate, branched hyphae. ***Conidiophores*** 145–360 µm long, 6.5–7.5 µm wide (x̄ = 210 × 6.5 μm, n = 20), macronematous, mononematous, cylindrical, unbranched or branched at base, straight to slightly bent, septate, deep brown at root part, brown at apex, pale brown at middle part mixing with some brown areas, smooth-walled with irregular inclusion. ***Conidiogenous cells*** 13–22 µm long, 5–7.5 µm wide (x̄ = 17 × 6.4 μm, n = 20), monoblastic, integrated, sympodial, terminal, cylindrical or fertile at the apex of conidiophores, brown, smooth-walled with irregular inclusion; with denticles, 1.3–2.3 µm long, 1.4–2.5 µm wide (x̄ = 1.6 × 1.8 μm, n = 20), arising from the apex portion of conidiophores as tooth-like and papillate protrusions, exposed or imbedded in the apex of conidiophore, mono- to polyblastic, brown, smooth-wall. ***Conidia*** 18–22.5 μm diam. (x̄ = 20.4 μm, n = 40) and conidial filament 6.8–9 μm wide (x̄ = 8.2 μm, n = 40), 45–55 μm long (x̄ = 50.6 μm, n = 40), solitary, acrogenous, helicoid, rounded at tip, tapering towards flat end, conic truncate at base, tightly coiled 1½ times, 8-septate, not becoming loose in water, guttulate, hyaline to pale brown, smooth-walled, the third cell shrinking and producing the root canal.

##### Culture characteristics.

***Conidia*** germinating on water agar and germ tubes produced from conidia within 12 h. ***Colonies*** growing on PDA attaining 2.5 cm diam. after six weeks at 25 °C, irregular, undulate, rough, superficial and partially immersed, brown aerial mycelium mixed with pale brown, deep brown at up and down junction area; reverse brown with pale brown.

##### Material examined.

China, Yunnan Province, Xishuangbanna City, an unidentified forest beside National Highway 219 (21°93'N, 101°24'E, 549.6 m elev.), rotting petiole of the *Elaeisoleifera*, 5 February 2023, Y.R. Xiong and Li Lu, XG198 (MHZU 23-0157, holotype); ex-type culture, ZHKUCC 24-0121, other living cultures ZHKUCC 24-0122, ZHKUCC 24-0766, ZHKUCC 24-0767.

##### Notes.

**Figure 2.**
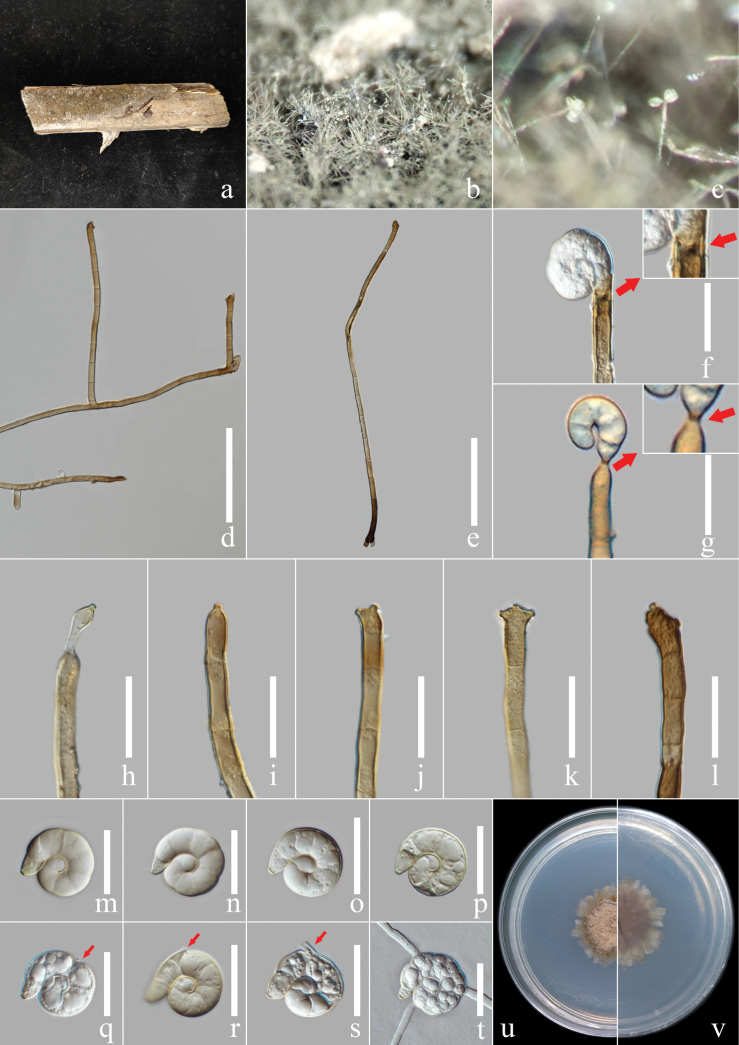
*Helicomaoleifera* (MHZU 23-0157, holotype) **a** specimen observed **b, c** colony on decaying *Elaeisoleifera***d, e** conidiophores **f, g** conidiogenous cell with attached conidia **h–l** conidiogenous cells **m–p** conidia **q–s** conidia produce the tubular structure at the third cell **t** germinated conidium **u, v** culture on PDA from above and reverse. Scale bars: 100 μm (**d, e**); 20 μm (**f–t**).

Four isolates obtained in this study from the rotting petiole of the *Elaeisoleifera* clustered in an independent clade in the phylogenetic tree with 78% ML, 76% MP bootstrap support and 1.00 BIPP bootstrap support. The nucleotide differences between *Helicomaoleifera* and its phylogenetically related species were checked, excluding gaps: *H.acropleurogenum* (GZCC 22-2035) - ITS: 3.53% (18/510 base pairs), LSU: 0.71% (6/844 base pairs), *tef* 1-*α*: 2.85% (26/912 base pairs), *rpb*2: 3.92% (41/1045 base pairs); *H.dennisii* (NBRC 30667) - ITS: 4.36% (25/573 base pairs), LSU: 0.35% (2/564 base pairs), *tef* 1-*α* and *rpb*2 sequence unavailable; *H.hydei* (MFLUCC 18-1270) - ITS: 3.50% (26/744 base pairs), LSU: 0.71% (6/847 base pairs), *tef* 1-*α*: 2.74% (25/912 base pairs), *rpb*2 sequence is unavailable; *H.inthanonense* (MFLUCC 11-0003) - ITS: 4.56% (26/570 base pairs), LSU: 1.63% (14/860 base pairs), *tef* 1-*α* and *rpb*2 sequence is unavailable. *Helicomaoleifera* is different from related species not only in the size of conidia and conidiophores (Table 3), but also in conidia, which shrink and produce the tubular structure at the third cell (Fig. 2q, r, s), while other species do not produce any deformation. In addition, *H.acropleurogenum* (Lu et al. 2023) has intercalary and mostly monoblastic, rarely polyblastic conidiogenous cells; however, *H.oleifera* has terminal and monoblastic or polyblastic conidiogenous cells. *Helicomadennisii* (Tsui et al. 2006) has intercalary and polyblastic conidiogenous cells and fertile denticle structure at several cells on the upper end of the conidiophore. However, *H.oleifera* only has fertile denticle structures at the apex cell of the conidiophore. Furthermore, *H.oleifera* differs from *H.hydei* (Liu et al. 2019) by having an embedded denticle structure, while *H.hydei* (Liu et al. 2019) has an exposed denticle structure. Furthermore, *H.inthanonense* (Boonmee et al. 2011) has acropleurogenous and brown conidia with 7-septate and produces an asexual morph from MEA culture, while *H.oleifera* has acrogenous and hyaline to pale brown conidia with 8-septate. Therefore, we introduce *H.oleifera* as a new species.

**Table 3.** Comparison of asexual morph characteristics of *Helicoma* species in this study; the names of strains in this study are indicated in bold.

Species names and culture accession numbers	Conidiophores	Conidiogenous cells	Conidia	Septate number	Colour	Coiled times	References
*Helicomaacropleurogenum* GZCC 22-2035	118–389 μm long, 5.5–8.5 μm wide (x̄ = 219 × 6.5 μm, n = 20)	20–32 μm long, 5–8 μm wide (x̄ = 25 × 6 μm, n = 20)	21–24 μm diam. and conidial filament 8.5–10.5 μm wide (x̄ = 22.0 × 9.5 μm, n = 20), 48–58 μm long	6–7	pale brown	tightly coiled 1½–1¾ times	Lu et al. (2023)
*Helicomainthanonense* MFLUCC 11-0003	(14.5–)26.5–34(−42) μm in diam., 3 μm wide	NA	(10–)13–20 μm in diam., 4–7 μm wide (x̄ = 14 × 6 μm)	7	hyaline to brown	NA	Boonmee et al. (2011)
*Helicomahydei* MFLUCC 18-1270	135–310 μm long, 4.5–7.0 μm wide	13–37 μm long, 4.5–7.0 μm wide	19–30 μm diam. (x̄ = 25.0 μm, n = 20), conidial filament 6–12 μm wide (x̄ = 8.1 μm, n = 20)	NA	pale brown to brown	tightly coiled 1–1½ times	Liu et al. (2019)
*Helicomadennisii* NBRC 30667	3.5–5 µm wide at the basal part and tapering to 3–3.5 µm wide at the apical part, up to 190 µm long	1–1.5 × 0.5–1 µm	10–15 (13.5) µm in diam.; conidial filament hyaline to dilute fuscous, 4–5.5 (4.5) µm thick	6–9 (8)	Hyaline	tightly coiled 1¼–1¾ (1½) times	Zhao et al. (2007)
***Helicomaoleifera*ZHKUCC 24-0121**	145–360 μm long, 6.5–7.5 μm wide (x̄ = 235 × 6.8 μm, n = 20)	13–22 μm long, 5–7.5 μm wide, tiny tooth–like protrusions (1.3–2.3 μm long, 1.4–2.5 μm wide)	18–22.5 μm diam. and conidial filament 6.8–9 μm wide (x̄ = 20.4 μm diam., 8.2 μm wide, n = 50), 45–55 μm long	8	pale brown to brown	tightly coiled 1½ times	This study
*Helicomaguttulatum* MFLUCC 16-0022	74–182 (197) μm long, 4–6 μm wide (x̄ = 120 × 5 μm, n = 20)	NA	18–23 μm diam. and conidial filament 6–8 μm wide (x̄ = 20 × 7 μm, n = 20)	8–9	hyaline to pale brown	tightly coiled 1–1½ times	Hyde et al. (2016)
***Helicomaguttulatum*ZHKUCC 24-0139**	75–225 μm long, 5.5–6 μm wide (x̄ = 152 × 5.7 μm, n = 20)	10–29 μm long, 5–8.8 μm wide, tiny tooth–like protrusions (1.6–3.5 μm long, 1.6–2.5 μm wide)	21–30 μm diam. and conidial filament 7.2–10 μm wide (x̄ = 25 μm diam., 8.4 μm wide, n = 50), 48–69 μm long	8–9	pale brown	tightly coiled 1½ times	This study
*Helicomarufum* MFLUCC 17-1806	110–210 μm long, 7–8.5 μm wide	9–14 μm long, 5.5–8.5 μm wide, tiny tooth–like protrusions (2.5–3.6 μm long, 1.5–2 μm wide)	35–45 μm diam. and conidial filament 4–5.5 μm wide (x = 41 × 4.5 μm, n = 20), 240–410 μm long	27–37	hyaline to pale brown	coiled 2–3 times, becoming loosely coiled in water	Lu et al. (2018b)
***Helicomarufum*ZHKUCC 24-0143**	150–270 µm long, 4–7.5 µm thick (x̄ = 225 × 5.9 μm, n = 20)	7–15 μm long, 4–7 μm wide, tiny tooth–like protrusions (3–6 μm long, 1.5–3 μm wide)	21–47 μm diam. and conidial filament 2–5 μm wide (x̄ = 36 × 3.8 μm, n = 40), 145–345 μm long	25–35	hyaline	tightly coiled 3–4.5 coils	This study
*Neohelicosporiumhyalosporum* GZCC 16-0076	up to 540 μm long, 4–5.5 μm wide	9–13 μm long, 4–5.5 μm wide	25–33 μm diam. and conidial filament 3–4 μm wide (x̄ = 28 μm diam., 3.5 μm wide, n = 50), 125–225 μm long	NA	hyaline	tightly coiled 2.5–3.5 times, becoming loosely coiled in water	Lu et al. (2018a)
*Neohelicosporiumovoideum* GZCC 16-0064	up to 420 μm long, 4–6 μm wide	10–15 μm long, 4–6 μm wide	25–35 μm diam. and conidial filament 3–4 μm wide (x̄ = 28 × 3.5 μm, n = 50), 180–230 μm long	multi–septate	hyaline	tightly coiled 2–3 times, becoming loosely coiled in water	Lu et al. (2018b)
***Neohelicosporiumguineensis*ZHKUCC 24-0113**	50–160 μm long, 4–6 μm wide (x̄ = 120 × 5.2 μm, n = 10)	11.5–20 μm long, 3.5–5.5 μm wide, tiny tooth–like protrusions (1.4–2.7 μm long, 1.2–2 μm wide)	16–20 μm diam. and conidial filament 1.8–3 μm wide (x̄ = 18 μm diam., 2.4 μm wide, n = 50), 90–130 μm long	11–12	hyaline	tightly coiled 2½–3½ times, loosely coiled in water	This study
*Neohelicosporiumfusisporum* MFUCC 16-0642	NA	12–20 μm long, 1.5–2.5 μm wide	18–22 μm diam. and conidial filament 1.5–2.5 μm wide (x̄ = 18 μm × 2 μm, n = 50), 100–150 μm long	multi–septate	hyaline	tightly coiled 2½–3¼ times, loosely coiled in water	Jayasiri et al. (2017)
***Neohelicosporiumxishuangbannaensis*ZHKUCC 24-0119**	40–125 μm long, 3–6 μm wide (x̄ = 68.4 × 4.4 μm, n = 20)	7–14 μm long, 2.5–5.5 μm wide, tiny tooth–like protrusions (1.8–3.3 μm long, 1.1–2.3 μm wide)	16.5–20.5 μm diam. and conidial filament 1.8–3.2 μm wide (x̄ = 18.5 μm diam., 2.4 μm wide, n = 50), 90–125 μm long	9–13	hyaline	tightly coiled 2–3¼ times, loosely coiled in water	This study

#### ﻿ Helicoma guttulatum

Taxon classificationFungiTubeufialesTubeufiaceae

Y.Z. Lu, Boonmee & K.D. Hyde, Fungal Diversity 80: 1–270 (2016)

6F3CF67D-C90E-5336-83AA-04A62CD9C50D

Index Fungorum: IF552218

Facesoffungi Number: FoF02358

Fig. 3

##### Description.

***Saprobic*** on the rotting petiole of *Caryotamitis*. ***Sexual morph***: Not observed. ***Asexual morph***: Hyphomycetous, helicosporous. ***Colonies*** on the substratum superficial, effuse, gregarious, brown. ***Mycelium*** composed of partly immersed, partly superficial, brown, septate hyphae. ***Conidiophores*** 75–225 μm long, 5.5–6 μm wide (x̄ = 152 × 5.7 μm, n = 20), macronematous, crowded, erect, straight to slightly bent, brown, deep brown towards the base, septate, branched, smooth-walled with irregular inclusion. ***Conidiogenous cells*** 10–29 μm long, 5–8.8 μm wide (x̄ = 18 × 6.3 μm, n = 20), mono- to polyblastic, integrated, cylindrical, terminal, pale brown to brown, smooth-walled with irregular inclusion; with denticles, 1.6–3.5 μm long, 1.6–2.5 μm wide (x̄ = 2.4 × 2.1 μm, n = 20), arising from the apex portion of conidiophores as tooth-like protrusions, mono- to polyblastic, brown, smooth-wall. ***Conidia*** 21–30 μm diam. (x̄ = 25.1 μm, n = 40) and conidial filament 7.2–10 μm wide (x̄ = 8.4 μm, n = 40), 48–69 μm long (x̄ = 57.4 μm, n = 40), solitary, acropleurogenous, tightly coiled 1½ times, guttulate, not becoming loose in water, hyaline to pale brown, tapering towards flat end, 8–9-septate, rounded at the apex, conic truncate at the base, smooth-walled.

**Figure 3.**
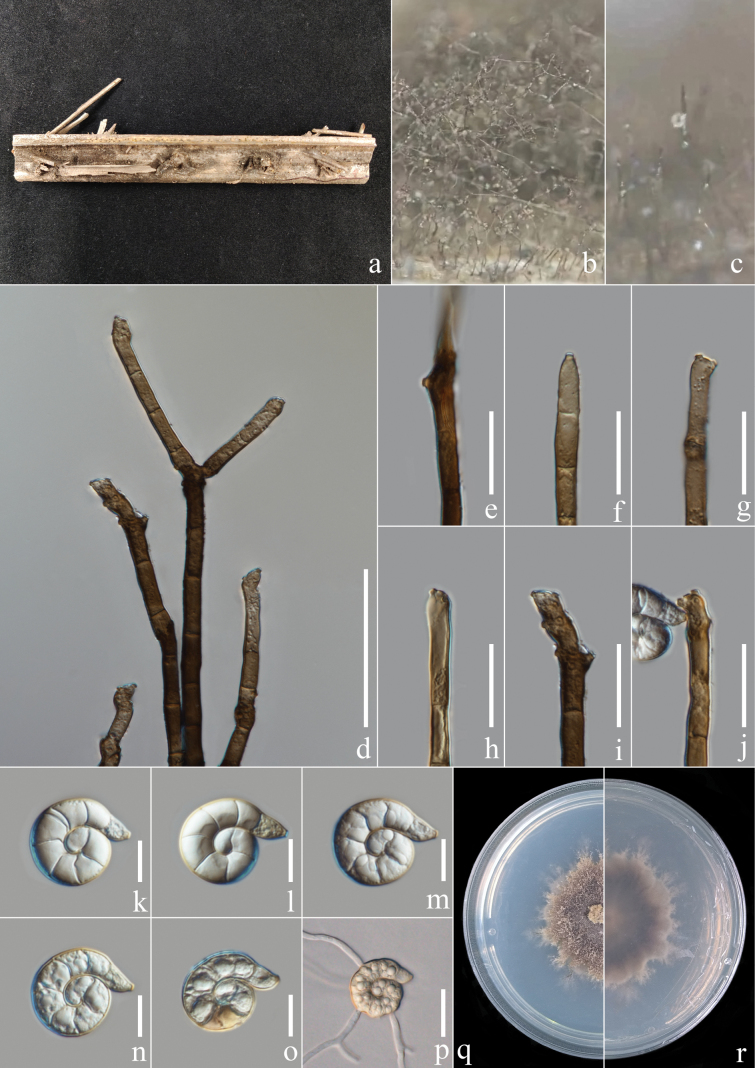
*Helicomaguttulatum* (MHZU 23-0166, new host record) **a** specimen observed **b, c** colony on decaying *Caryotamitis***d** conidiophores **e–j** conidiogenous cells, thereinto **j** with attached conidia **k–o** conidia **p** germinated conidium **q, r** culture on PDA from above and reverse. Scale bars: 50 μm (**d**); 20 μm (**e–j**); 10 μm (**k–o**); 20 μm (**p**).

##### Culture characteristics.

***Conidia*** germinating on water agar and germ tubes produced from conidia within 12 h. ***Colonies*** growing on PDA attaining 3 cm diam. after six weeks at 25 °C, irregular, undulate, rough, superficial and partially immersed, brown aerial mycelium mixed with pale brown; reverse brown with pale brown.

##### Material examined.

China, Yunnan Province, Xishuangbanna City, an unknown forest beside National Highway 219 (21°93'N, 101°24'E, 549.6 m elev.), rotting petiole of the *Caryotamitis*, 5 February 2023, Y.R. Xiong and Li Lu, XG215 (MHZU 23-0166, new host record; living culture, ZHKUCC 24-0139, ZHKUCC 24-0140).

##### Notes.

Two isolates on rotting petiole of the *Caryotamitis* obtained in this study clustered with the *H.guttulatum* clade, based on the phylogenetic tree with 100% ML, 100% MP bootstrap support and 0.91 BIPP bootstrap support. The nucleotide differences excluding gaps between *H.guttulatum* (ZHKUCC 24-0139) and *H.guttulatum* (MFLUCC 16-0022) in ITS is 3.60% (17/472 base pairs), while there is no difference in LSU and one base pair difference in *tef* 1-*α* and *rpb*2. Our two isolates are similar to *H.guttulatum* (Hyde et al. 2016) in shape, colour and size of conidia (Table 3). Although the conidiophores are longer than in previous collections, this might be due to the branching of the conidiophore of the isolates in this study, whereas the previous collections are unbranched. In addition, the location of the denticles is the same. Therefore, based on morphology and phylogenetic analysis, we identified our isolates as *H.guttulatum* and this is a new record of *H.guttulatum* on *Caryotamitis*. *Helicomaguttulatum* was first introduced on decaying wood from Thailand by Hyde et al. (2016), based on morphology and phylogeny. Tian et al. (2022) reported a new collection of *H.guttulatum* on decaying wood of an unidentified host from Thailand.

#### ﻿ Helicoma rufum

Taxon classificationFungiTubeufialesTubeufiaceae

Y.Z. Lu, J.C. Kang & K.D. Hyde, Fungal Diversity 92: 131–344 (2018)

94D30D65-FAF0-5F56-9DEC-78E0E3658540

Index Fungorum: IF554843

Facesoffungi Number: FoF04718

Fig. 4

##### Description.

***Saprobic*** on the rotting inflorescence of *Caryotamitis*. ***Sexual morph***: Not observed. ***Asexual morph***: Hyphomycetous, helicosporous. ***Colonies*** on the substratum superficial, effuse, gregarious, pale brown. ***Mycelium*** composed of partly immersed, partly superficial, hyaline to brown, septate, branched hyphae. ***Conidiophores*** 150–270 µm long, 4–7.5 µm wide (x̄ = 225 × 5.9 μm, n = 20), macronematous, mononematous, cylindrical, erect, straight to slightly bent, pale brown to deep brown from top towards the base, apex hyaline, septate, mostly unbranched, smooth-walled. ***Conidiogenous cells*** 7–15 μm long, 4–7 μm wide (x̄ = 12 × 5.9 μm, n = 20), mono- to polyblastic, cylindrical, integrated, intercalary, brown, smooth-walled; with denticles, 3–6 μm long, 1.5–3 μm wide (x̄ = 4.6 × 2.5 μm, n = 20), arising from the lower portion of conidiophores as tooth-like protrusions, mono- to polyblastic, pale brown to brown, smooth-walled. ***Conidia*** 21–47 μm diam. (x̄ = 36.2 μm, n = 40) and conidial filament 2–5 μm wide (x̄ = 3.8 μm, n = 40), 145–345 μm long (x̄ = 257.7 μm, n = 40), solitary, pleurogenous, tightly coiled 3–4½ times, guttulate, become loose in water, hyaline to pale brown, 25–35-septate, smooth-walled.

**Figure 4.**
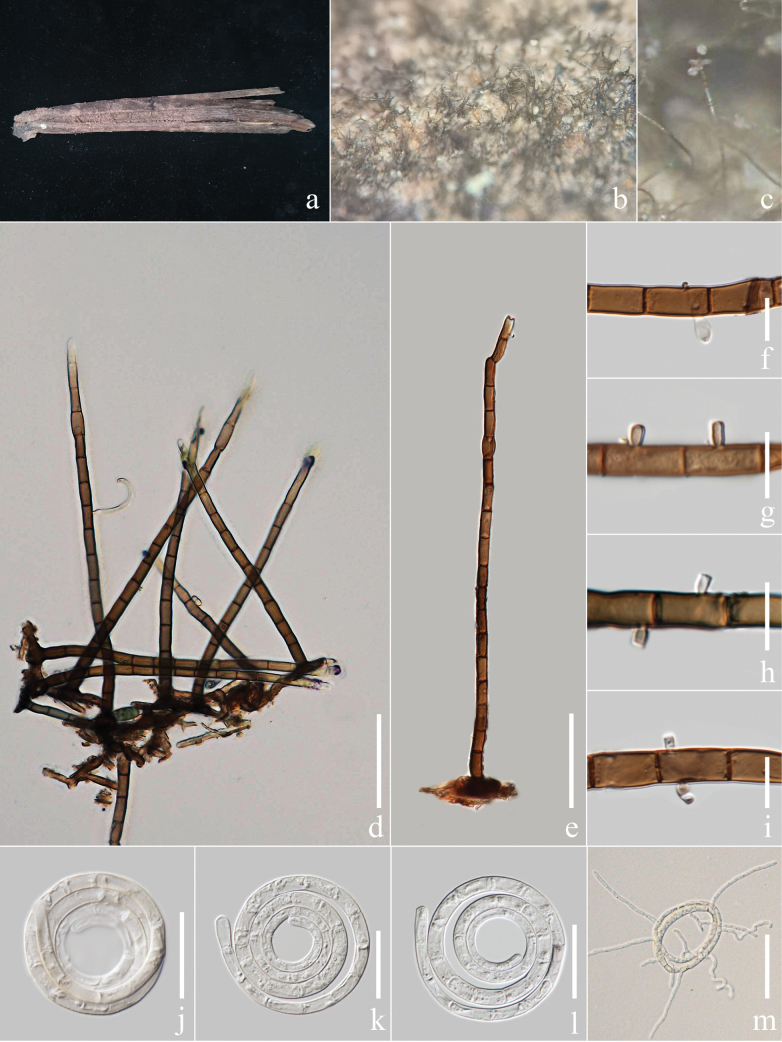
*Helicomarufum* (MHZU 23-0168, new host record) **a** specimen observed **b, c** colony on decaying *Caryotamitis***d, e** conidiophores **f–i** conidiogenous cells **j–l** conidia **m** germinated conidium. Scale bars: 50 μm (**d, e, m**); 10 μm (**f–i**); 20 μm (**j–l**).

##### Culture characteristics.

***Conidia*** germinating on water agar and germ tubes produced from conidia within 12 h. ***Colonies*** growing on PDA attaining 3 cm diam. after six weeks at 25 °C, irregular, undulate, rough, superficial and partially immersed, brown aerial mycelium mixed with pale brown; reverse brown with pale brown.

##### Material examined.

China, Yunnan Province, Xishuangbanna City, an unidentified forest beside National Highway 219 (21°93'N, 101°24'E, 549.6 m), rotting inflorescence of the *Caryotamitis*, 5 February 2023, Y.R. Xiong and Li Lu, XG217 (MHZU 23-0168, new host record; living culture, ZHKUCC 24-0143, ZHKUCC 24-0144).

##### Notes.

Two isolates on rotting inflorescence of *Caryotamitis* obtained in this study clustered with the *H.rufum* clade in the phylogenetic tree with 96% ML, 95% MP bootstrap values and 0.99 BIPP bootstrap support. The nucleotide differences between *H.rufum* (ZHKUCC 24-0143) and *H.rufum* (MFLUCC 17-1806) are LSU: 0.09% (1/1171 base pairs), *tef* 1-*α*: 0.22% (2/912 base pairs), *rpb*2 sequence unavailable and no difference in ITS, excluding gaps. Our collection is similar to *H.rufum* (Lu et al. 2018b) in the shape, colour and size of conidia (Table 3). Although the conidiophores and conidia are longer than in previous collections, this might be because the collections came from a different area, resulting in branching at the top of the conidiophores. Therefore, based on phylogenetic and morphological analysis, we identified our isolates as a new host record of *H.rufum* on *Caryotamitis*. *Helicomarufum* was introduced from decaying wood in Thailand by Lu et al. (2018b), based on the distinguished phylogenetic clade, wider conidiophores and larger tooth-like conidiogenous protrusions and larger conidia.

#### ﻿ Neohelicosporium guineensis

Taxon classificationFungiTubeufialesTubeufiaceae

Y.R. Xiong, Manawas. & K.D. Hyde sp. nov.

FA246873-BAD4-5372-98D3-136CF07F0C97

Index Fungorum: IF902154

Facesoffungi Number: FoF15912

Fig. 5

##### Etymology.

Species epithet refers to the host species name “*guineensis*” from which the fungus was isolated.

##### Holotype.

MHZU 23-0153.

##### Description.

***Saprobic*** on the rotting petiole of *Elaeisguineensis*. ***Sexual morph***: Not observed. ***Asexual morph***: Hyphomycetous, helicosporous. ***Colonies*** on the substratum superficial, effuse, gregarious, brown. ***Mycelium*** composed of partly immersed, partly superficial, pale brown, glistening, septate, branched hyphae. ***Conidiophores*** 50–160 µm long, 4–6 µm wide (x̄ = 120 × 5.2 μm, n = 20), macronematous, mononematous, cylindrical, unbranched or branched at apex, straight, septate, pale brown, brown at root part, smooth-walled. ***Conidiogenous cells*** 11.5–20 µm long, 3.5–5.5 µm wide (x̄ = 15.5 × 4.8 μm, n = 20), mono- to polyblastic, integrated, sympodial, terminal or intercalary, cylindrical, yellowish to pale brown, smooth-walled; with denticles, 1.4–2.7 µm long, 1.2–2 µm wide (x̄ = 1.9 × 1.6 μm, n = 20), arising from the juncture portion of two conidiogenous cells as tooth-like protrusions, mono- to polyblastic, hyaline, smooth-walled. ***Conidia*** 16–20 μm diam. (x̄ = 18 μm, n = 40) and conidial filament 1.8–3 μm wide (x̄ = 2.4 μm, n = 40), 90–130 μm long (x̄ = 112.9 μm, n = 40), solitary, mostly pleurogenous, rarely acrogenous, helicoid, rounded at tip, obvious hump and constricted at septa, coiled 2½–3½ times, 11–12-septate, becoming loose in water, guttulate, hyaline, smooth-walled.

**Figure 5.**
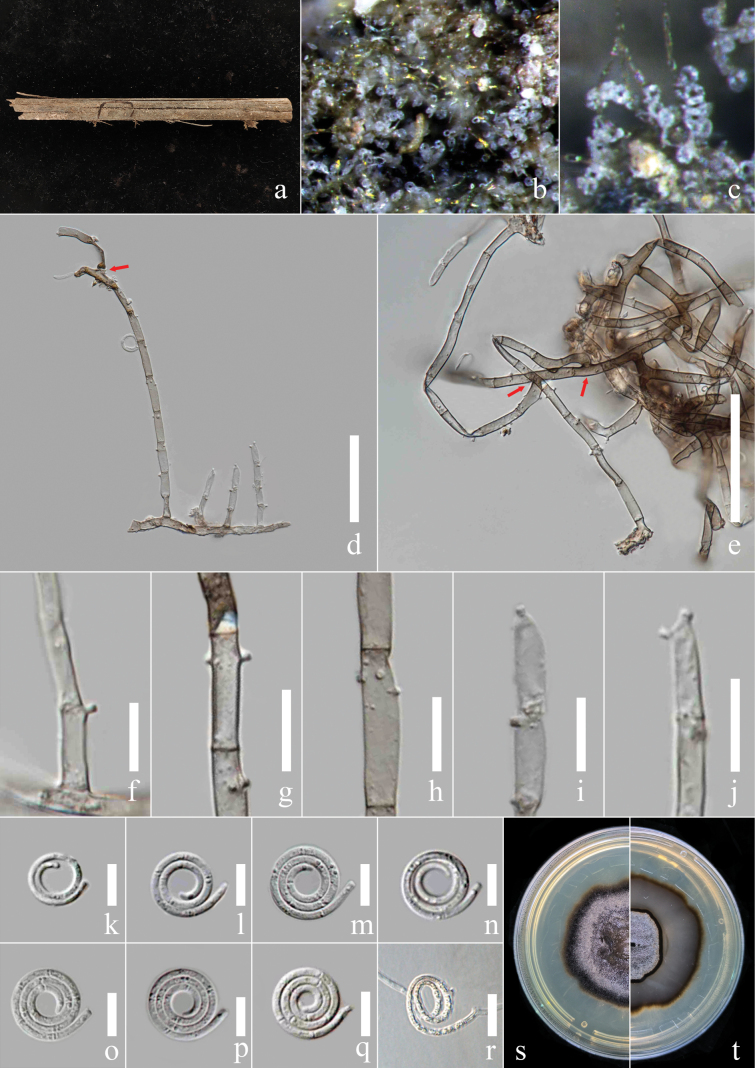
*Neohelicosporiumguineensis* (MHZU 23-0153, holotype) **a** specimen observed **b, c** colony on decaying *Elaeisguineensis***d, e** apical branches forming long connected conidiophores **f–j** conidiogenous cells **k–q** conidia **r** germinated conidium **s, t** culture on PDA from above and reverse. Scale bars: 50 μm (**d, e**); 10 μm (**f–q**); 20 μm (**r**).

##### Culture characteristics.

***Conidia*** germinating on water agar and germ tubes produced from conidia within 12 h. ***Colonies*** growing on PDA attaining 3.5 cm diam. after six weeks at 25 °C, irregular, undulate, umbonate, rough, superficial and partially immersed, white aerial mycelium, deep brown at immersed area; reverse white to deep brown.

##### Material examined.

China, Yunnan Province, Xishuangbanna City, an unidentified forest beside National Highway 219 (21°93'N, 101°24'E, 549.6 m elev.), rotting petiole of the *Elaeisguineensis*, 5 February 2023, Y.R. Xiong and Li Lu, XG186 (MHZU 23-0153, holotype); ex-type, ZHKUCC 24-0113, other living culture ZHKUCC 24-0114.

##### Notes.

Two isolates from this study formed a separate lineage and clustered with *Neohelicosporiumhyalosporum* and *N.ovoideum* in the phylogenetic tree with 88% ML, 78% MP bootstrap support and 1.00 BIPP bootstrap support. The nucleotide differences excluding gaps between *N.guineensis* and its phylogenetically related species were checked: *N.hyalosporum* (GZCC 16-0076) - ITS: 1.56% (8/513 base pairs), LSU: 0.83% (7/840 base pairs), *tef* 1-*α*: 1.32% (12/912 base pairs), *rpb*2: 3.63% (38/1045 base pairs); *N.ovoideum* (GZCC 16-0064) - ITS: 1.50% (8/534 base pairs), LSU: 0.48% (4/826 base pairs), *tef* 1-*α*: 1.21% (11/912 base pairs), *rpb*2: 3.16% (33/1045 base pairs). *Neohelicosporiumguineensis* differs from its closely-related species in the size of conidia and conidiophores (Table 3). *Neohelicosporiumhyalosporum* and *N.ovoideum* are multi-septate and are not constricted at the septa, while *N.guineensis* are 11–12-septate and constricted at the septa (Lu et al. 2018a, b). *Neohelicosporiumhyalosporum* (Lu et al. 2018a) has multi-denticles in one conidiogenous cell, while *N.guineensis*, has no more than three denticles (Fig. 5e, f) in one conidiogenous cell. Furthermore, *N.ovoideum* (Lu et al. 2018b) has 1–2 short-connecting cells between conidiophores, while *N.guineensis* has one long connecting cell (Fig. 5d, e) which connects conidiophores at the apex. Based on the phylogenetic placement and morphological variations, we introduce *N.guineensis* as a new species.

#### ﻿ Neohelicosporium xishuangbannaensis

Taxon classificationFungiTubeufialesTubeufiaceae

Y.R. Xiong, Manawas., & K.D. Hyde sp. nov.

67B51B3E-4B98-5EAA-A79B-8E24337D3C23

Index Fungorum: IF902156

Facesoffungi Number: FoF15913

Fig. 6

##### Etymology.

Species epithet refers to the location name “Xishuangbanna” from where the holotype was collected.

##### Holotype.

MHZU 23-0156.

##### Description.

***Saprobic*** on the rotting petiole of *Elaeisguineensis*. ***Sexual morph***: Not observed. ***Asexual morph***: Hyphomycetous, helicosporous. ***Colonies*** on the substratum superficial, effuse, gregarious, brown. ***Mycelium*** composed of partly immersed, partly superficial, brown, septate, unbranched hyphae. ***Conidiophores*** 40–125 μm long, 3–6 μm wide (x = 68.4 × 4.4 μm, n = 20), macronematous, mononematous, flexuous, long, cylindrical, branched, septate, smooth-walled. ***Conidiogenous cells*** 7–14 μm long, 2.5–5.5 μm wide (x̄ = 11.2 × 3.9 μm, n = 20), mono- to polyblastic, integrated, sympodial, terminal or intercalary, cylindrical, pale brown, smooth-walled; with denticles, 1.8–3.3 μm long, 1.1–2.3 μm wide (x̄ = 2.4 × 1.4 μm, n = 20), arising from the juncture portion of two conidiogenous cells as tooth-like and papillate protrusions, mono- to polyblastic, pale brown or hyaline, smooth-walled. ***Conidia*** 16.5–20.5 μm diam. (x̄ = 18.5 μm, n = 40) and conidial filament 1.8–3.2 μm wide (x̄ = 2.4 μm, n = 40), 90–125 μm long (x̄ = 107 μm, n = 40), solitary, acropleurogenous, helicoid, rounded at tip, coiled 2–3¼ times, 9–13-septate, becoming loose in water, guttulate, slightly constricted at septa, hyaline to pale brown, smooth-walled.

##### Culture characteristics.

***Conidia*** germinating on water agar and germ tubes produced from conidia within 12 h. ***Colonies*** growing on PDA attaining 2.5 cm diam. after six weeks at 25 °C, irregular, undulate, umbonate, rough, superficial and partially immersed, brown aerial mycelium mixed with pale brown, deep brown at up and down junction area; reverse brown with deep brown.

##### Material examined.

China, Yunnan Province, Xishuangbanna City, an unidentified forest beside National Highway 219 (21°93'N, 101°24'E, 549.6 m elev.), rotting petiole of the *Elaeisguineensis*, 5 February 2023, Y.R. Xiong and Li Lu, XG197 (MHZU 23-0156, holotype); ex-type, ZHKUCC 24-0119, other living culture ZHKUCC 24-0120.

##### Notes.

**Figure 6.**
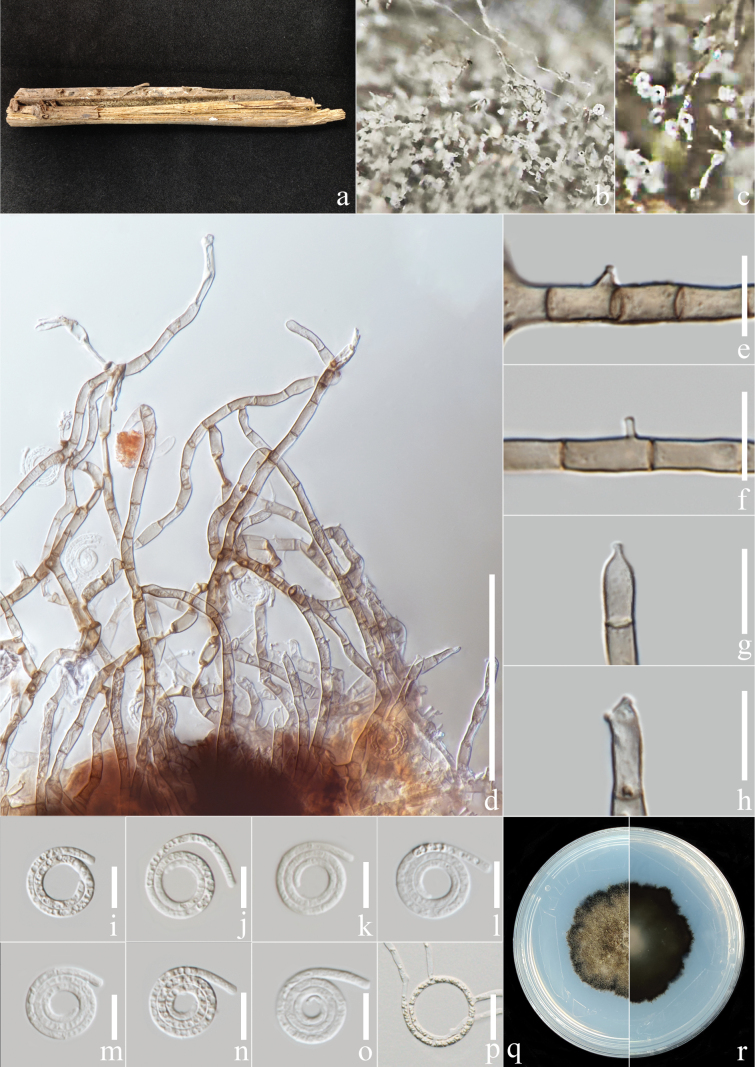
*Neohelicosporiumxishuangbannaensis* (MHZU 23-0156, holotype) **a** specimen observed **b, c** colony on decaying *Elaeisguineensis***d** conidiophores **e, f** intercalary conidiogenous cells **g, h** terminal conidiogenous cells **i–o** conidia **p** germinated conidium **q, r** culture on PDA from above and reverse. Scale bars: 50 μm (**d**); 10 μm (**e–o**); 20 μm (**p**).

Two isolates obtained in this study developed an independent clade in the phylogenetic tree with 77% ML, 79% MP bootstrap support and 0.99 BIPP bootstrap support. The nucleotide differences excluding gaps between *Neohelicosporiumxishuangbannaensis* and *N.fusisporum* (MFUCC 16-0642) are ITS: 2.81% (15/533 base pairs), LSU: 1.06% (9/852 base pairs), *tef* 1-*α*: 2.41% (22/912 base pairs) and *rpb*2 sequence is unavailable. *Neohelicosporiumfusisporum* was reported as a sexual and asexual morph by Jayasiri et al. (2017). *Neohelicosporiumxishuangbannaensis* is different from the asexual morph of *N.fusisporum* (Jayasiri et al. 2017) in the size of conidia and conidiogenous cells (Table 3). In addition, the asexual morph of *N.fusisporum* (Jayasiri et al. 2017) has an intercalary conidiogenous cell, while *N.xishuangbannaensis* has an intercalary (Fig. 6e, f) or terminal (Fig. 6g, h) conidiogenous cell. Furthermore, the asexual morph of *N.fusisporum* (Jayasiri et al. 2017) has denticles with tooth-like or long neck cells, while *N.xishuangbannaensis* has a denticle with tooth-like and papillate protrusions. Based on these differences, herein we introduce *N.xishuangbannaensis* as a new species.

## ﻿Discussion

In the present study, we identified and introduced three new species viz. *Helicomaoleifera*, *Neohelicosporiumguineensis* and *N.xishuangbannaensis* with two new host records of *Helicoma* viz. *H.guttulatum* and *H.rufum*, which are associated with palms in tropical China. Xishuangbanna forests comprise numerous palm species, including *Caryota* sp., *Calamus* sp. and *Elaeis* sp. This humid tropical area near streams is also an ideal environment for Tubeufiaceae species (Lu et al. 2018b). In addition, most previous reports of this family are on unknown decaying woods (Lu et al. 2023) and we believe that there may be more undiscovered records of Tubeufiaceae on palms in tropical regions. Furthermore, in our comparison with closely-related species, we observed that *H.anastomosanse* (David 1931), *H.divaricatum* (Holubová-Jechová 1987) and *H.westonii* (David 1931) were reported to inhabit palms, but no molecular data were available for conducting phylogenetic analysis. The spores of *H.anastomosanse* (David 1931) have 18–25 septa, *H.westonii* (David 1931) have 11–14 septa and *H.divaricatum* (Holubová-Jechová 1987) have branched conidiophores and pleurogenous spores, which can be clearly distinguished from *H.oleifera*. However, the lack of molecular data for the above-mentioned three species has posed a significant challenge, forcing us to spend more time on morphological comparisons to identify *H.oleifera*. Similarly, almost all Tubeufiaceae reported on palm hosts in the early 20^th^ century lack molecular data, which further complicates our task of sorting out the information on Tubeufiaceae on palm hosts.

*Helicoma* is one of the most typical helicosporous genera (Lu et al. 2023), although Goos (1986) and Lu et al. (2018b) successively revised this genus. In addition, the species of this genus cluster on the same large branch in phylogenetic analysis; some morphologically similar species are in different subordinate clades. In addition, we observed that *Helicomaguttulatum* (ZHKUCC 24-0139), which was identified, based on phylogenetic analysis has a different morphology compared to the type (Hyde et al. 2016). *Helicomaguttulatum* (ZHKUCC 24-0139) was observed to have branched conidiophores and is different from *H.guttulatum* (MFLU 21-0183) unbranched conidiophores (Tian et al. 2022). Since the two collections were collected from different locations and climates, we hypothesise that isolations could be influenced by different locations and climates. However, further collections and detailed analysis are required to confirm this hypothesis.

*Neohelicosporium* was introduced to accommodate helicosporous taxa with distinct conidiophores and is supported by molecular phylogenies, based on ITS, LSU, *tef* 1-*α* and *rpb*2 sequence data (Lu et al. 2018a). However, the bootstrap values of ML and MP are below 0.75% for some species (e.g. *N.abuense*, *N.astrictum* and *N.bambusicola*) within this genus (Lu et al. 2018a; Tian et al. 2022). The three new species and two new host records introduced in this study are significant as they expand our understanding of the diversity and distribution of Tubeufiaceae in tropical regions and provide valuable insights into their ecological roles and interactions with palm hosts. In addition, Zhang et al. (2023) identified four useful chemical compounds from *N.guangxiense* and they can play a vital role in drug design and functional group modification. This underscores the urgent need for future studies to explore the potential chemical composition and corresponding applications of this genus, a call to action for professional researchers.

## Supplementary Material

XML Treatment for Helicoma oleifera

XML Treatment for Helicoma guttulatum

XML Treatment for Helicoma rufum

XML Treatment for Neohelicosporium guineensis

XML Treatment for Neohelicosporium xishuangbannaensis

## ﻿Additional information

### Author ORCIDs

Yinru Xiong https://orcid.org/0000-0002-4673-606X

Kevin D. Hyde https://orcid.org/0000-0002-2191-0762

Li Lu https://orcid.org/0000-0003-0977-6414

Dulanjalee L. Harishchandra https://orcid.org/0000-0003-1538-4951

Ausana Mapook https://orcid.org/0000-0001-7929-2429

Ishara S. Manawasinghe https://orcid.org/0000-0001-5730-3596

### Data availability

All of the data that support the findings of this study are available in the main text.
